# The Effect of Glucose and Poloxamer 188 on Red-Blood-Cell Aggregation

**DOI:** 10.3390/metabo11120886

**Published:** 2021-12-18

**Authors:** Alicja Szołna-Chodór, Bronisław Grzegorzewski

**Affiliations:** Department of Biophysics, Collegium Medicum in Bydgoszcz, Nicolaus Copernicus University, ul. Jagiellońska 15, 85-067 Bydgoszcz, Poland; alicja.szolna@cm.umk.pl

**Keywords:** RBC aggregation, glucose, poloxamer 188

## Abstract

Glucose metabolism disorders contribute to the development of various diseases. Numerous studies show that these disorders not only change the normal values of biochemical parameters but also affect the mechanical properties of blood. To show the influence of glucose and poloxamer 188 (P188) on the mechanical properties of a red-blood-cell (RBC) suspension, we studied the aggregation of the cells. To show the mechanisms of the mechanical properties of blood, we studied the effects of glucose and poloxamer 188 (P188) on red-blood-cell aggregation. We used a model in which cells were suspended in a dextran 70 solution at a concentration of 2 g/dL with glucose and P188 at concentrations of 0–3 g/dL and 0–3 mg/mL, respectively. RBC aggregation was determined using an aggregometer, and measurements were performed every 4 min for 1 h. Such a procedure enabled the incubation of RBCs in solution. The aggregation index determined from the obtained syllectograms was used as a measure of aggregation. Both the presence of glucose and that of P188 increased the aggregation index with the incubation time until saturation was reached. The time needed for the saturation of the aggregation index increased with increasing glucose and P188 concentrations. As the concentrations of these components increased, the joint effect of glucose and P188 increased the weakening of RBC aggregation. The mechanisms of the observed changes in RBC aggregation in glucose and P188 solutions are discussed.

## 1. Introduction

The metabolic diseases plaguing today’s civilization result from damage to normal metabolisms, especially in cases such as diabetes mellitus, in which the carbohydrate pathway is disrupted by inappropriate insulin secretion or abnormal responses by the body. With this disruption of the carbohydrate pathway, there is an elevated level of glucose in the blood, called hyperglycemia. The glucose consumption of most cells is regulated by insulin. Red blood cells (RBCs) do not need insulin; however, in the absence of this hormone, they are exposed to high glucose levels during hyperglycemia. Glucose has an effect on RBCs, changing their biochemical properties and the ability of these cells to aggregate. Changes in these processes depend on the period of time the cells are exposed to glucose and on the glucose concentration, but also on the composition of the solution in which the RBCs are suspended. Polymers are widely known among the substances that change the aggregation of RBCs. Thus, it is particularly important to understand the simultaneous effects of glucose and drug polymers on RBC aggregation.

Biochemical tests, which are the basis for the diagnosis of diabetes, are increasingly supplemented with analyses of the rheological properties of blood [1,2]. Many factors influence the rheology of whole blood, including RBC aggregation. One of the first documented studies on the changes in RBC aggregation caused by elevated glucose levels was conducted in 1956 [3]. The in vivo effect of increased RBC aggregation under hyperglycemia was confirmed in the following years using various research methods and under various experimental conditions [4,5,6,7,8]. It has been shown that increased aggregation in patients with elevated glucose levels in type 2 DM decreases after the application of glycemic control, and this effect is visible both for RBCs suspended in autologous plasma and cells suspended in dextran solutions [8]. In general, the incubation of RBCs in glucose solutions in vitro causes a decrease in the aggregation of the cells. In order to demonstrate the effect in vitro, cells of healthy volunteers incubated in high glucose concentrations were studied [9,10,11,12]. RBC aggregation, as measured by the Aggregate Shape Parameter (ASP) method after two hours of incubation [10] and by the Myrenne method after half an hour of incubation [11], showed a downward trend.

In the case of blood, RBCs are suspended in the plasma, which may contain medicinal substances. One of these substances may be the nonionic surfactant poloxamer 188 (P188), which has many uses as a pharmaceutical excipient [13,14,15]. Extensive research is ongoing into its potential use as a biological membrane sealant [16,17,18], while other research is focused on its hemorheological effects [19,20,21,22]. In vitro and in vivo studies of the influence of P188 on hemorheological parameters in patients with acute myocardial infarction showed that the aggregation of RBCs in autologous plasma decreased after the application of this surfactant [19]. Conversely, the aggregation of RBCs of healthy volunteers measured in dextran 70 solution after in vitro treatment with P188 decreased, and the effect was dependent on the concentration of P188 [21]. The in vitro activity of P188 tested in dextran 70 solution was confirmed by studies of cells from healthy donors and donors with sickle cell anemia [18]. Another study showed a reduction in RBC deformability by 10% only after 2 h of incubation in P188 [22]. The determination of the influence of P188 on the aggregation of RBCs from healthy donors in dextran 70 solution, used as a medium for cells, allows for the exclusion of the effects of interindividual differences in plasma composition.

Knowledge of the possible joint effect of P188 and glucose is important for both the development of dosage forms and the prediction of enhancing effects in therapeutic applications that are still under development. There are studies investigating the synergistic effect of P188 and tinidazole on the solubility and drug release profile [23] or on the treatment of certain diseases [24], but the effect on RBC aggregation is a new area of research. Knowledge about the possible additional enhancement of the anti-aggregation effect of P188 is important, especially in light of reports about the need to administer P188 as soon as possible in order to support endogenous cell repair systems [25]. In this work, the effect of P188 on RBC aggregation in the presence of glucose was investigated. We present the results of a new approach where RBC aggregation was tested during the incubation of cells in dextran solutions with glucose and with or without P188. The aggregation of RBCs is usually measured immediately after sample preparation. In this article, we use a working hypothesis that the aggregation of RBCs changes during the incubation of these cells in solutions containing glucose and P188. Thus, we propose the measurement of temporal changes in RBC aggregation. This working hypothesis may be useful as the basis for further research into the effects of various substances on erythrocyte aggregation.

## 2. Results

**Figure 1:**
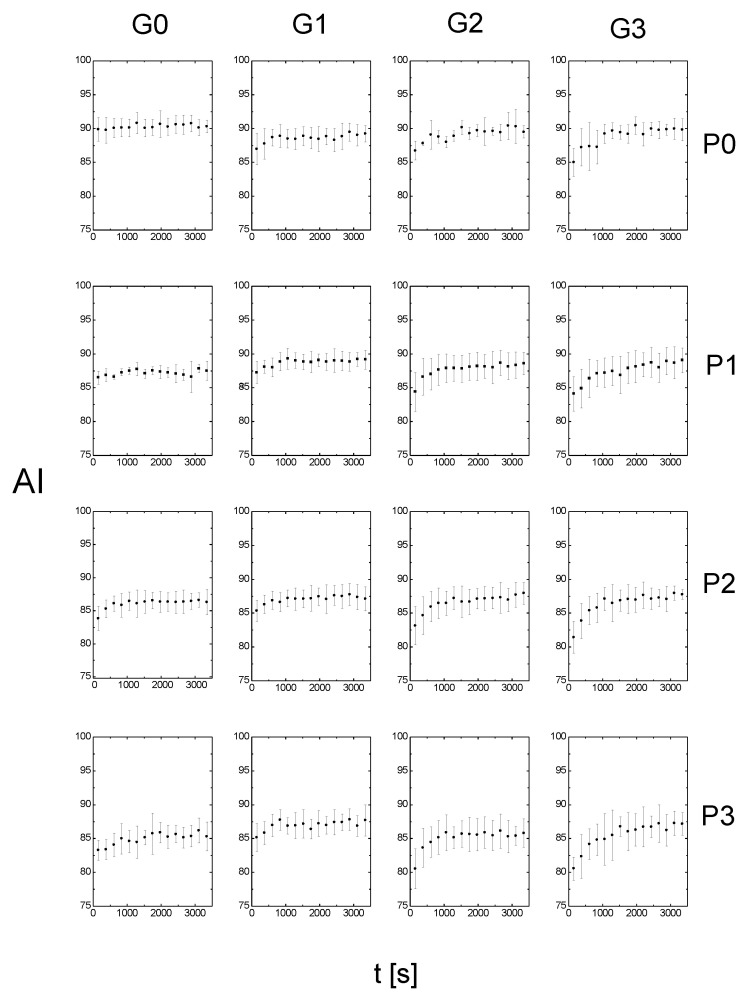
Aggregation index (AI) as a function of time for RBCs incubated in a dextran solution with P188 with concentrations ranging from 0 to 3 mg/mL (P1–P3) and glucose with concentrations ranging from 0 to 3 g/dL (G1–G3).

For each of the solutions used, the RBC aggregation index was measured based on the time. Figure 1 shows the data for the 16 solutions used in which RBCs were suspended. For every solution, six samples were prepared, and the results shown in Figure 1 represent the mean values of the aggregation index from the samples; the error bars represent the standard deviations. 

The first result (in the top left corner) shows data for RBCs suspended in dextran only. The first row contains the results for RBCs suspended in solutions with increasing concentrations of glucose. The next rows contain results for solutions containing P188 with increasing concentrations of glucose. The first column contains results for RBCs suspended in solutions with increasing P188 concentrations. The following columns contain results for glucose solutions with increasing P188 concentrations. As can be observed, the first result is the only one for which the aggregation index did not change over time. In the other cases, the aggregation index increased and became saturated over time. This saturation reflects a certain equilibrium state achieved by the system. The time it took for the system to reach equilibrium varied with the concentration of glucose and P188. In addition, depending on the concentrations of glucose and P188, the starting and ending values of the aggregation index changed. 

**Figure 2:**
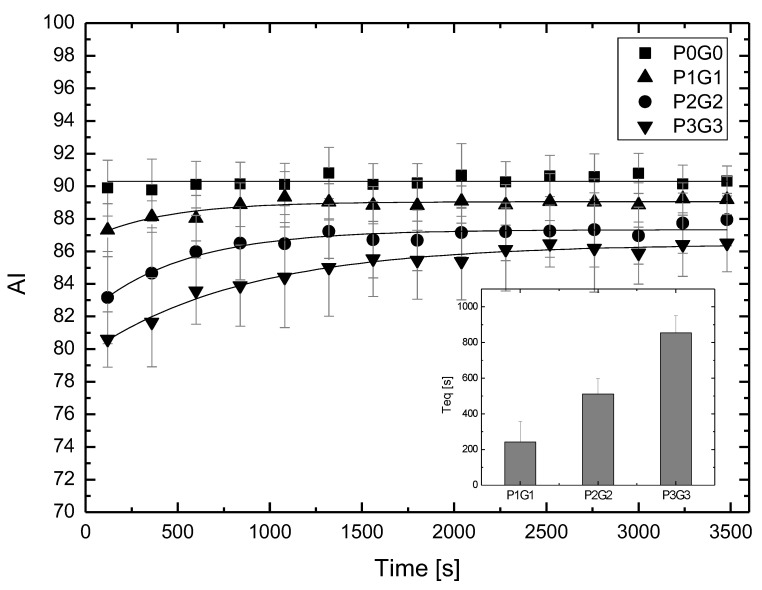
The aggregation index (AI) as a function of incubation time. P0G0 means RBCs suspended in dextran 70 only. P1G1 denotes addition of P188 at a concentration of 1 mg/mL and glucose at 1 g/dL. P2G2 and P3G3 represent the higher concentrations of P188 and glucose. The fits were made to obtain the Teq parameter. In the inset of this figure, the dependence of the time necessary to reach an equilibrium by the system Teq on the concentrations of P188 and glucose is shown.

As can be seen from Figure 1, the changes in the aggregation parameters are particularly visible for the results distributed on the diagonal. Figure 2 shows these results together with the fit, allowing the time taken for the system to reach equilibrium to be determined. The inset in Figure 2 shows how the time needed to reach equilibrium, Teq, increased as the concentration of glucose and P188 increased. Note that this equilibrium time increased both for RBCs suspended only in glucose solutions (row 1 in Figure 1) and for RBCs suspended only in P188 solutions (column 1 in Figure 1).

**Figure 3:**
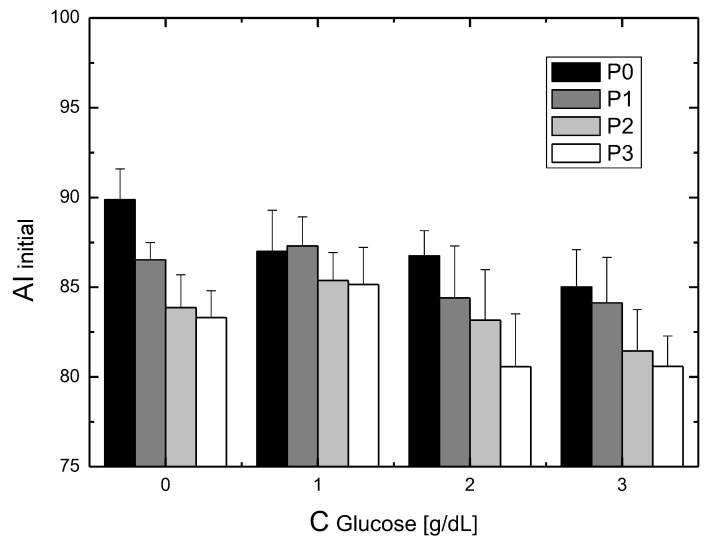
The dependence of the aggregation index (AI), obtained for the first measurement during incubation (2 min after the start of the experiment), on the concentration of glucose and P188. A decrease of the initial aggregation index with glucose concentration is observed. The presence of P 188 causes an additional decrease of the aggregation index.

The data presented in Figure 1 make it possible to determine the initial aggregation index corresponding to the first measurement and the final aggregation index corresponding to the last measurement in a given series. This initial aggregation index for the RBCs suspended in the test solutions is shown in Figure 3. The column groups contain the results for the specific glucose values and, for each glucose value, the values for the different P188 concentrations. In general, it can be seen that an increase in glucose concentration resulted in a decrease in the initial aggregation index. The addition of P188 exacerbated this effect, causing a further reduction in the initial aggregation index. Except for the results for a glucose concentration of 1 g/dL, in all the other cases, the addition of increasing concentrations of P188 resulted in a decrease in the initial aggregation index values. 

**Figure 4:**
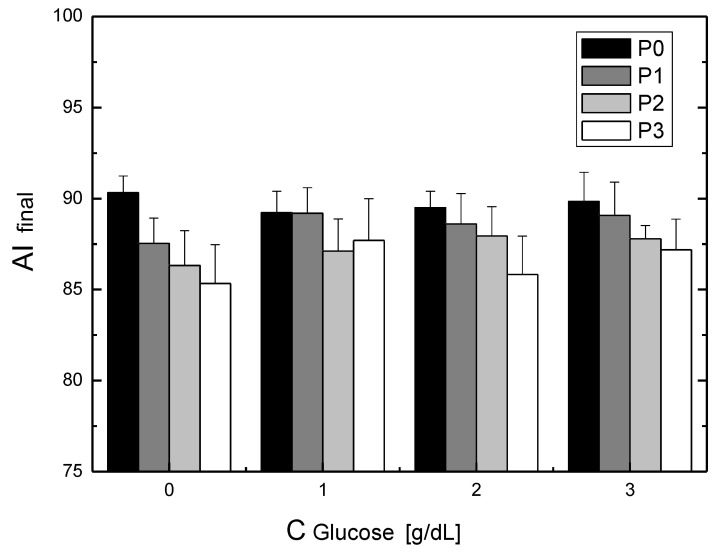
The dependence of the aggregation index (AI) obtained at 1 h of the incubation on the concentration of glucose and P188. The presence of P 188 causes a decrease of the aggregation index.

The final aggregation index for the RBCs suspended in the test solutions is shown in Figure 4. As previously stated, the column groups contain the results for the specific glucose values and, for each glucose value, the values for the different P188 concentrations. It can be seen that the addition of P188 caused a reduction in the final aggregation index. As previously stated, except for the results for a glucose concentration of 1 g/dL, in all the other cases, the addition of increasing concentrations of P188 resulted in a decrease in the final aggregation index values. 

## 3. Discussion

Metabolic processes are time dependent, and their courses are modified by the presence of drugs and other substances in the body. Both of these elements are taken into account in this article. The subject of this study was the time dependence of RBC aggregation parameters in solutions containing glucose and the influence of P188 added to these solutions on the aggregation parameters. Thus, in addition to the temporal effect, the joint effect of glucose and P188 on RBC aggregation was investigated.

Studying the influence of glucose on the rheological properties of blood has a long history and results from the need to understand the mechanisms involved in diabetes [26]. Research shows that incubation of RBCs in glucose solutions causes oxidation of membrane lipids and glycation of proteins, which leads to a reduction of RBC aggregation [9,12]. Shin et al. suggest that these changes occur even after 1 h of incubation. We conduct research depending on time. Thus, oxidation and glycation affect changes in RBC aggregation; however, the results presented in this paper suggest that glucose transport to erythrocytes plays a key role in the temporal changes in the aggregation of these cells. Elevated blood glucose also affects red-blood-cell aggregation, but the in vivo and in vitro effects are not the same and are still not fully understood; thus, this issue still needs to be investigated [3,4,5,6,7,8,9,10,11]. The results presented in this study show that, for RBCs placed in glucose solutions, the aggregation index increases with the time of incubation in these solutions. After the growth period, the saturation of this parameter is observed. The time required for saturation increases as the glucose concentration in the solution increases. The aggregation index values measured immediately after placing the RBCs in solutions containing glucose decrease with the concentration of glucose. However, the aggregation index measured at the time of saturation does not show such a marked decrease as a function of the glucose concentration. This means that the time of the incubation of the RBCs in the glucose solution has a significant influence on the course of aggregation and may lead to differing results [9,10,11,12].

The phenomenon of transport occurs for RBCs placed in glucose solutions. Glucose enters the RBCs, and this transport does not require the presence of insulin [27]. As a result, the concentration of glucose in the solution decreases while the concentration of glucose inside the RBCs increases. Lowering the glucose concentration in the solution in which the RBCs are suspended results in a lower viscosity of the solution [28,29]. This happens until equilibrium is achieved—when the transportation back and forth is the same. The study of this transport showed that the values of the parameters of this transport significantly differ depending on whether the transport takes place inside the RBC or in the solution [12]. This immediately indicates that the issue of the incubation time and cell cleaning after incubation can cause major changes in aggregation parameters. Finally, a question arises about the mechanisms of the change in aggregation during incubation in glucose solutions. Research shows that the biochemical changes of RBCs incubated in glucose solutions occur slowly. Thus, it can be assumed that, for the results shown here, the biochemical changes had little effect on the changes in aggregation. This allows for a hypothesis that, in the cases considered here, mechanical changes in the cell membrane and changes in the viscosity of the substance in which the RBCs are dissolved determine the changes in the aggregation of these cells.

For RBCs placed in P188 solutions, as in the case of RBCs placed in solutions containing glucose, we observed an increase in the aggregation index with the incubation time. The saturation effect was also observed. In this case, both the initial and final aggregation index values decreased with increasing P188 concentrations in the solution. This RBC aggregation behavior corresponding to the initial aggregation index values studied here was observed using the microscopic aggregation index (MAI) and Myrenne method [21]. As in the previous case, a question arises about the mechanism of the aggregation process in the case of RBCs in solutions containing P188. The reduction in the aggregation index appears to be due to the presence of a low-molecular-weight polymer in the solution. According to the depletion theory of aggregation, such polymers reduce the aggregation capacity of RBCs [30]. The question remains open, however, of why the presence of polaxamer causes temporary changes in the RBC aggregation index similar to the temporal changes in the aggregation index of RBCs placed in glucose solutions.

Consider the problem of the synergistic effect of glucose and P188 on RBC aggregation. According to the definition, a synergistic effect takes place when the effect of processes interacting together is greater than the effects of the individual processes. The results presented in this study show that the presence of P188 enhances the process of suppressing glucose-dependent RBC aggregation. We showed this effect, expected by clinicians, only in vitro. The mechanism of lowering aggregation in this case results from the aggregation mechanisms for glucose solutions and the aggregation mechanism for P188 solutions. It is not certain, however, whether the mechanism of the synergistic effect is a simple assembly of partial mechanisms.

Each measurement technique has certain limitations. This is also the case for RBC aggregation measurements. When choosing the measurement technique, we were guided by the need to simultaneously measure the aggregation and incubation of cells. In addition, we were looking for a technique that would allow for relatively fast repeatable measurements. The choice fell on a photometric technique that meets the above-mentioned requirements. The approach proposed in this paper enables RBC aggregation measurements with a resolution of 4 min.

In conclusion, we performed a study of the effects of P188 in the presence of glucose on RBC aggregation. The aggregation was studied as a function of time. The study showed a synergistic effect of glucose and P188 on RBC aggregation and the temporary changes in this aggregation. To the best of our knowledge, the synergistic effect of P188 and glucose, as well as temporal changes, on aggregation are presented for the first time. The research method applied and the results obtained appear to be promising for further research in this area.

## 4. Materials and Methods

### 4.1. Materials 

Human venous blood was obtained from 96 healthy adult volunteers. The blood was collected in sterile tubes containing the K3-EDTA anticoagulant and was maintained at 4 °C until processing, which was performed as soon as possible. The RBCs were separated from whole blood via centrifugation at 3000× *g* rpm for 5 min at 4 °C; next, the buffy coat and plasma were discarded. The fractionated RBCs were washed three times with phosphate-buffered saline (PBS), pH 7.4, under the same centrifugation conditions as before. Dextran with a molecular mass of 70 kDa (Dextran from *Leuconostoc* spp., Sigma-Aldrich, MO, USA), glucose (D-(+)-Glucose, Sigma-Aldrich, MO, USA) and P188 (Poloxamer 188 Solid, Alfa Aesar, TX, USA) were used to make the solutions in PBS. The sixteen solutions for RBCs were prepared, in which dextran was always at the same concentration, while glucose and P188 were combined at different concentrations. The final concentrations were as follows: dextran: 2 g/dL; glucose: 0, 1, 2 or 3 g/dL; and P188: 0, 1, 2 or 3 mg/mL. The RBCs were suspended in these solutions immediately before measurement. The hematocrit of these suspensions was adjusted to 40%. The measurements were performed at room temperature (22 ± 1 °C). All the experiments were performed according to the guidelines of the Bioethics Commission of Collegium Medicum Nicolaus Copernicus University.

### 4.2. Method

**Figure 5:**
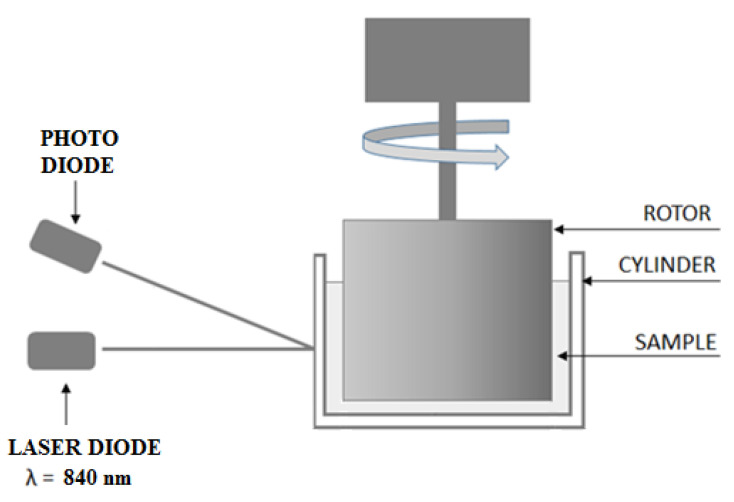
Experimental set up.

RBC aggregation was determined using an aggregometer, as shown in Figure 5. The investigated suspension was placed in a transparent cylinder with an internal diameter of 32 mm. A rotor with an outer diameter of 30.9 mm was placed in the cylinder. The layer of the suspension was illuminated by laser diode light at a wavelength of 840 nm. The backscattered light was detected by a photodiode, and the backscattered light’s intensity was recorded as a function of time.

**Figure 6:**
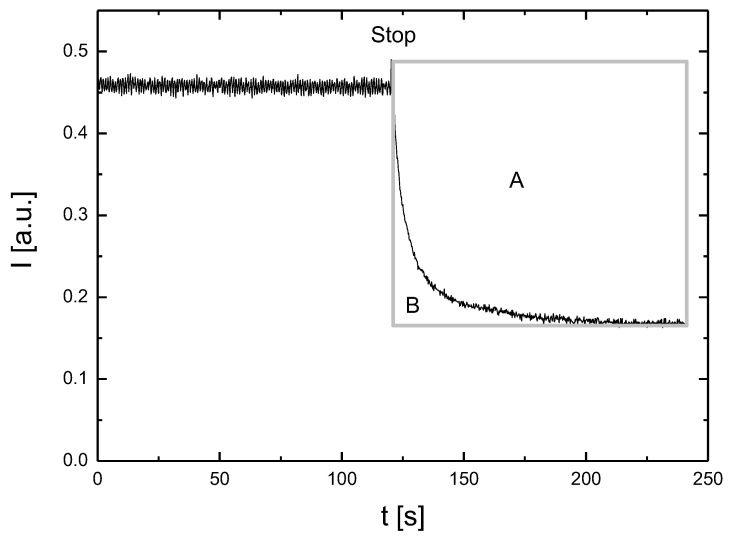
Syllectogram of RBC aggregation in dextran.

The measurement was carried out for one hour. During this time, the rotor was operated in cycles of 2 min of rotation and 2 min of rest. Figure 6 shows the light intensity of the backscattered light for the first cycle for RBCs suspended in dextran. During the first part of the cycle, the intensity remained constant. Due to the rotor operation, the RBC suspension was subjected to a shear stress at a shear rate of 165 s^−1^. During the first part of the cycle, red blood cells were completely disaggregated and deformed through elongation. Stopping the rotor first caused the light intensity to increase rapidly and then to slowly decrease. The rapid increase in light intensity reflects the return of the RBCs to their original shape. The decrease in light intensity was the result of RBC aggregation. The light intensity recorded in this last part of the cycle was recorded as a syllectogram. The aggregation index AI = 100 × A/(A + B) was taken as a measurement of RBC aggregation, where surfaces A and B are shown in Figure 6.

**Figure 7:**
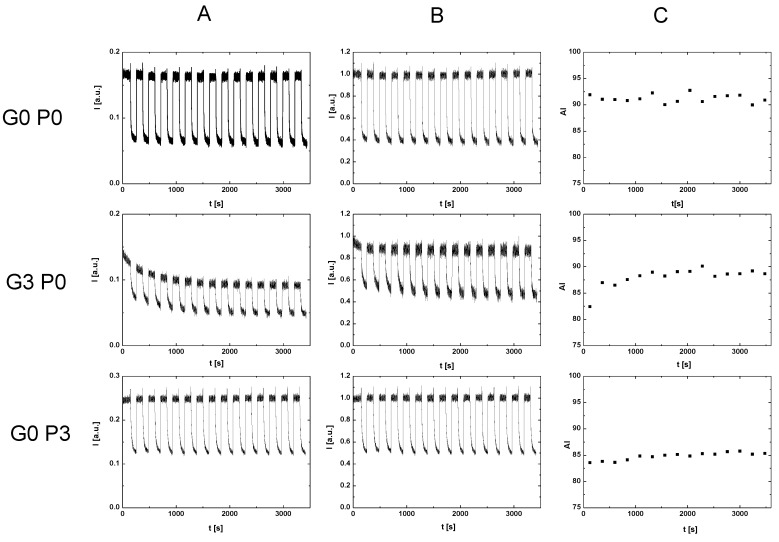
Scheme of data analysis. Column (**A**) shows the intensity obtained from the measurement. Column (**B**) shows the normalized intensity, and column (**C**) contains the values of the aggregation index over time. The first row represents RBCs suspended in dextran, the second row represents RBCs suspended in glucose solution, and the last row represents RBCs suspended in P188.

The classical measurement of RBC aggregation was carried out on the basis of the first rotor cycle. In this paper, we present a new approach to measuring RBC aggregation by tracking this process during 15 rotor cycles. The procedure for measuring the time dependence of aggregation is shown in Figure 7. The first column in this figure shows the light intensity recorded over 1 h, sequentially, for RBCs suspended in dextran, for RBCs suspended in glucose solution, and for RBCs suspended in P188 solution. As can be observed, the upper light intensity envelope was constant for RBCs suspended in dextran and for RBCs suspended in P188 solution, while it changed with time for RBCs suspended in glucose solution. For this reason, the light intensity in all cases was divided by the intensity value corresponding to the upper envelope. In this way, the data presented in the second column were obtained. Based on these data, the aggregation index was determined for each syllectogram. This resulted in time-dependent aggregation index values. These values are presented in the third column. The presented method makes it possible to determine the aggregation index with a resolution of 4 min. An additional advantage of this method is that, during the measurement, RBCs were incubated in the solution in which they were suspended, and the effect of this incubation on aggregation could be recorded. 

## Data Availability

The data presented in this study are available on request from the corresponding author according to local policies.
